# Human Claudin-7 *cis*-Interactions Are Not Crucial for Membrane-Membrane (*Trans*-) Interactions

**DOI:** 10.3389/fmolb.2022.908383

**Published:** 2022-06-27

**Authors:** Lena Ahlswede, Carmen Siebenaller, Benedikt Junglas, Nadja Hellmann, Dirk Schneider

**Affiliations:** ^1^ Department of Chemistry, Biochemistry, Johannes Gutenberg University Mainz, Mainz, Germany; ^2^ Institute of Molecular Physiology, Johannes Gutenberg University Mainz, Mainz, Germany

**Keywords:** claudin, tight junction, heterologous expression, proteoliposomes, atomic force microscopy, membrane protein, oligomerization

## Abstract

Human Claudin-7 (Cldn7) is a member of the Claudin (Cldn) superfamily. *In vivo*, these proteins form tight junctions, which establish constricted connections between cells. Cldns oligomerize within the membrane plane (= *cis*-interaction), and also interact with Cldns from adjacent cells (= *trans*-interaction). Interactions of Cldns are typically studied *in vivo* and structural analyses of isolated Cldns are limited. Here, we describe heterologous expression in *E. coli* and purification of human Cldn7, enabling *in vitro* analyses of the isolated protein using detergent and model membrane systems. Cldn7 exists as a monomer, hexamer, and various higher oligomers in micelles. While only limited unfolding of the protein was observed in the presence of the anionic detergent sodium dodecyl sulfate, decreased ionic strength did affect Cldn7 *cis*-interactions. Furthermore, we identified two amino acids which mediate electrostatic *cis*-interactions and analyzed the impact of disturbed *cis*-interaction on *trans*-contacts via atomic force microscopy and monitoring Förster resonance energy transfer between fluorescently labeled Cldn7-containing proteoliposomes. Our results indicate that Cldn7 *cis*-oligomerization might not be a prerequisite for establishing *trans*-contacts.

## 1 Introduction

The interaction of tight junction-forming protein complexes from two neighboring cells tightly links individual epithelial or endothelial cells. Thereby, they control the paracellular transport of molecules (Overgaard et al., 2011). Furthermore, tight junction-forming proteins assemble into strands within a cell´s plasma membrane, polarizing individual cells (Zihni et al., 2016) to separate an apical from a basolateral plasma membrane region (Lu et al., 2013). While the formation of tight junctions typically involves several proteins, including occludins or cytoskeleton-linking scaffold proteins that link the cytoskeleton to the tight junction (*e.g.,* ZO-1, ZO-2), the core tight junction components are claudins (Cldns) (Chiba et al., 2008; González-Mariscal et al., 2008). In fact, the expression of individual Cldns alone results already in intramembrane assembly of Cldn strands and the formation of tight junctions (Furuse et al., 1998).

The Cldn superfamily involves 27 members in mammals (Mineta et al., 2011), albeit only 23 Cldns are present in humans (Lal-Nag and Morin, 2009). Cldns are small α-helical transmembrane (TM) proteins with a molecular mass of 20–35 kDa (Liu et al., 2016). All Cldns possess four TM helices (Van Itallie and Anderson, 2006; Günzel and Yu, 2013), with both the N- and the C-terminus being localized within the cytoplasm (Furuse et al., 1998). While the N-terminus is typically only a few amino acids long, the C-terminus is variable in length (Lal-Nag and Morin, 2009) and establishes interactions with other tight junction proteins (Itoh et al., 1999). A large extracellular loop 1 (ECL 1) connects TM helices 1 and 2, and a second small extracellular loop 2 (ECL 2) connects TM helices 3 and 4 (Tsukita and Furuse, 2000). The two extracellular loops form the β-sheet headgroup of Cldn proteins, which can interact with Cldns from an adjacent cell for cell-cell linkage. Within the membrane plane, monomeric Cldns interact laterally (*cis*-interaction) for the formation of Cldn strands (Angelow et al., 2008), which then interact in *trans* for formation of tight junctions. While some Cldns form a tight, essentially impermeable paracellular barrier (Overgaard et al., 2011), others form intercellular pores that exhibit a charge specificity, which is determined by amino acid side chains of ECL 1 (Van Itallie and Anderson, 2006). Based on the crystal structure of murine Claudin15 (Cldn15), residues involved in homo-oligomerization and *cis*-interaction of Cldn monomers have been identified (Suzuki et al., 2017). Yet, it is still unclear whether the strong *cis*-interaction of Cldn monomers, *i.e.* the formation of Cldn strands, is required for *trans*-interaction, *i.e.* for cell-cell-linkage.

Here we present a first analyzis of *cis*- and *trans*-interactions of human Claudin 7 (Cldn7), a 211 amino acid protein (22.4 kDa) that is expressed in the collecting duct of the kidney (Li et al., 2004), respiratory tract (Coyne et al., 2003), intestine (Xing et al., 2020), and epididymis (Inai et al., 2007) on the basolateral side of epithelial cells. Cldn7 oligomers form an ion-permeable inter-cellular pore, albeit the selectivity of this pore is still under debate, and evidence for both Cl^−^-(Hou et al., 2006) and Na^+^-conduction exists (Alexandre et al., 2005, 2007). Altered expression of Cldn7 is observed in various cancer types (Kominsky et al., 2003; Ouban and Ahmed, 2010; Dahiya et al., 2011; Lu et al., 2011; Tsujiwaki et al., 2015; Ono et al., 2016), yet the exact role of Cldn7 in cancer progression is still unclear. We show that *cis*-interactions of purified human Cldn7 proteins can be studied *in vitro*, and the two residues R81 and Y149 appear to be key for oligomerization. Furthermore, a mutant with a disturbed oligomerization propensity still interacts in *trans*, suggesting that Cldn7 *cis*-interaction, *i.e.* strand formation, is no prerequisite for the formation of tight junctions. The here described techniques and analyses now offer the possibility to study the structure, stability, assembly and activity of isolated human Cldns in detail in model membrane systems.

## 2 Materials and Methods

### 2.1 Cloning and Heterologous Expression of Cldn7

The human Cldn7 gene (UniProt O95471) was amplified via PCR using the plasmid RC200530 (OriGene Technologies, Rockville, MD, United States) as a template and subsequently cloned into the plasmid pET-His6_StrepII_TEV_LIC (Addgene, Watertown, MA, United States) upon restriction digestion with SspI and MluI. Plasmids expressing proteins with the amino acid mutations V70A, R81A, F148A, Y149A, E160A, F161A or * (= V70A, R81A, F148A, Y149A, E160A, and F161A) were generated via site-directed mutagenesis (Liu and Naismith, 2008) using the primers listed in Supplementary Table S1.

For protein expression, the respective plasmid was transformed into chemical competent *E. coli* Tuner (DE3) pLysS cells. The bacteria were grown on LB-agar (100 μg/ml ampicillin) for 17 h. 50 ml LB-medium (100 μg/ml ampicillin) was inoculated with a single colony and incubated at 37°C and 200 rpm overnight. Next, these cultures were added to 2 L of LB-medium (100 μg/ml ampicillin) and cultivated at 37°C and 150 rpm until an OD_600_ of 0.6–0.8 was reached. Protein expression was induced by addition of 0.5 mM isopropyl-ß-*D*-thiogalactopyranoside (IPTG). After cultivation at 150 rpm for ∼18 h at 20°C, the bacteria were harvested by centrifugation at 1700 *g* (10 min, 4°C). The bacteria were snap-frozen in liquid nitrogen and stored at −20°C until further use.

### 2.2 Purification of Cldn7

Cldn7 carrying an N-terminal His_6_-tag was purified via immobilized metal affinity chromatography (IMAC) using nickel-nitrilotriacetic acid (Ni^2+^-NTA)-agarose (Macherey-Nagel, Düren, and GER). Therefore, bacteria pellets from a 2 L expression culture were resuspended in 50 ml lysis buffer (50 mM Na_2_HPO_4_/HCl (pH 8), 300 mM NaCl, 10% glycerol (hereafter: p-buffer)), with addition of 50 µl protease inhibitor cocktail (P8849 from Sigma-Aldrich, St. Louis, MO, United States). The cells were lysed using a Potter-Elvehjem homogenizer followed by high-pressure homogenization (Microfluidizer LM20 (MFIC, Newton, MA, United States), 3 runs with 18,000 PSI). The lysate was cleared by centrifugation at 12,000 *g* (10 min, 4°C). The membrane fraction was prepared by subsequent ultracentrifugation at 117,000 g (1 h, 4°C). Membranes were resuspended in 50 ml p-buffer + 2% (w/v) *N,N*-Dimethyldodecan-1-amine N-oxide (LDAO, from Alfa Aesar, Karlsruhe, GER), and proteins were extracted for 1 h at 4°C. The solution was incubated with 2.5 ml equilibrated Ni^2+^-NTA agarose beads with gentle agitation on a rotational shaker at room temperature (RT) for 1 h. The beads were transferred to a 5 ml column and washed with 30 ml p-buffer + 5 mM dodecyl-β-D-maltosid (DDM from Roth, Karlsruhe, GER) in presence of 5 mM imidazole followed by a 50 ml 50 mM imidazole wash-step. The protein was eluted with 5 × 2 ml p-buffer (+5 mM DDM, + 500 mM imidazole). The buffer was exchanged to p-buffer (+5 mM DDM) by a PD10-desalting column (Macherey-Nagel, Düren, and GER). The protein concentration was determined photometrically by measuring the absorbance at 280 nm and using the calculated extinction coefficients listed in Supplementary Table S2 (Gasteiger et al., 2005). The solubilized proteins were snap-frozen in liquid nitrogen and stored at −20°C. For protein insertion into DOPC (1,2-dioleoyl-*sn*-glycero-3-phosphocholine) liposomes, the Ni-NTA purification was performed in p-buffer with 0.2% LDAO instead of 5% DDM. For analyses, the proteins were separated on 10% SDS-PAGE gels and stained with Coomassie brilliant blue R250 or analyzed via Western blotting. The proteins were detected using an antibody recognizing the His-tag (HisTag Antibody HRP conjugate from Merck Millipore, Darmstadt, GER).

### 2.3 Size Exclusion Chromatography

Size Exclusion Chromatography (SEC) was used to analyze the oligomeric state of Cldn7 with an ÄKTA basic system (GE Healthcare, Munich, GER) and a Superose12 10/300 GL column (GE Healthcare, Munich, GER) equilibrated with p-buffer (+5 mM DDM) at 8°C. Protein elution from the column was monitored via following the absorbance at 280 nm. The column was calibrated using standards of known size (blue dextran (>2000 kDa), β-amylase (200 kDa), albumin (67 kDa), carbonic anhydrase (29 kDa), cytochrome c (13 kDa) and Acetyl-CoA (0.8 kDa) from Sigma-Aldrich (St. Louis, MO, United States)).

### 2.4 Analysis of Solubilized Cldn7 in Mixed DDM/SDS Micelles

Increasing amounts of SDS were added to 3.2 μM purified Cldn7 dissolved in p-buffer (+5 mM DDM) in a total volume of 200 µl. The SDS mole fraction χ_SDS_ of the samples (Eq. 1) was stepwise increased from 0 to 0.95.

$$\chi_{\mathrm{SDS}}=\;\frac{c_{\mathrm{SDS}}}{c_{\mathrm{SDS}}+c_{\mathrm{DDM}}}$$ (1)

Here, c_SDS_ refers to the SDS concentration and c_DDM_ refers to the DDM concentration.

The samples were incubated for 1 h at RT prior to the fluorescence measurements.

### 2.5 Trp Fluorescence Measurements

Tryptophan fluorescence spectra were recorded at 25°C from 290 to 450 nm (slit 2 nm) in 1 nm steps upon excitation at 280 nm (slit 2 nm) using quartz crystal suprasil cuvettes (3 mm; Hellma Analytics, Jena, GER) on a Fluoromax-4 spectrometer (Horiba Scientific, Kyoto, JPN).

To analyze changes of the Trp fluorescence emission spectrum, the *average emission wavelength* <λ> (also described as *spectral center of mass*) was analyzed according to Eq. 2, which includes changes of the emission maximum, but also the spectral shape (Royer et al., 1993).

$$<\lambda >\;=\;\frac{\sum \lambda \cdot I}{\sum I}$$ (2)

Here, λ refers to the wavelength and I refers to the fluorescence intensity.

### 2.6 NaCl-Induced Destabilization of Cldn7 Oligomers

1 ml purified Cldn7 (∼4 µM) was dialyzed (Spectra/Por® MWCO 12–14 kDa from Roth, Karlsruhe, GER) against 3 × 1 L of p-buffer in presence of 0–300 mM NaCl. The solution was centrifuged at 20,000 g for 1 min at RT. A sample of the supernatant was prepared for SDS-PAGE analysis. The pellet was resuspended in 100 µl 1 × SDS sample buffer. For quantitative analysis, the monomer band of a Coomassie-R250-stained 12% SDS-PAGE gel was quantified using the program ImageJ, as described in (Schneider et al., 2012).

### 2.7 Reconstitution of Cldn7 Into DOPC Liposomes

DOPC (1,2-dioleoyl-*sn*-glycero-3-phosphocholine) and the fluorescently labeled lipids NBD-DOPE (1,2-dioleoyl-*sn*-glycero-3-phosphoethanolamine-N-(7-nitro-2-1,3-benzoxadiazol-4-yl) (ammonium salt) and Liss-Rhod-DOPE (Lissamine Rhodamine PE; 1,2-Dioleoyl-sn-glycero-3-phosphoethanolamine-N-(lissamine rhodamine B sulfonyl) (ammonium salt)) were purchased from Avanti Polar Lipids, Inc. (Birmingham, AL, United States). For liposome preparation, lipids were dissolved in chloroform. The organic solvent was evaporated under a gentle stream of nitrogen gas followed by vacuum desiccation overnight to remove remaining traces of solvent. Destabilized liposomes were prepared by hydration of the dried lipid film with p-buffer (+6 mM LDAO) and five cycles of freeze-thawing. For reconstitution of Cldn7, purified Cldn7 in p-buffer (+6 mM LDAO) (final concentration 4.8 µM) was mixed 1:400 with DOPC-liposomes (final lipid concentration 2 mM, 0.1% fluorescently labeled lipids) and subsequently dialyzed 3 times against p-buffer at 4°C (Spectra/Por® Biotech CE MWCO 20000 from Roth, Karlsruhe, GER) to remove the detergent. The proteoliposomes were centrifuged at 20,000 g for 1 min at RT to remove aggregates.

### 2.8 Förster Resonance Energy Transfer (FRET)

Proteoliposomes, containing the FRET dyes Liss-Rhod-PE or NBD-PE, were prepared as described above. The differently labeled liposomes were mixed and incubated for 7 h at RT. 10 µl samples were taken at different time points and mixed with 190 µl p-buffer. The fluorescence was recorded from 475–700 nm (slit 5 nm) upon excitation of the FRET donor dye NBD at 460 nm (slit 5 nm).

As Liss-Rhod was excited at 460 nm to some extent, the spectra of the mixed labeled liposomes were corrected by subtracting spectra of pure Liss-Rhod-containing liposomes upon excitation at 460 nm. Possible, differences in the acceptor concentration were corrected by normalization to the intensity observed upon direct excitation of the acceptor in the respective solution at 590 nm. The ratiometric FRET efficiency (E_rat_) was calculated using Eq. 3.

$$E_{\mathrm{rat}}=\;\frac{1}{1+\;\frac{I_{\mathrm{Donor}}}{I_{\mathrm{Acceptor}}}}$$ (3)

Here, I_Donor_ refers to the donor fluorescence intensity at 530 nm (emission maximum of NBD) and I_Acceptor_ refers to the acceptor fluorescence intensity at 590 nm (emission maximum of Liss-Rhod).

### 2.9 Atomic Force Microscopy (AFM)

AFM was used to analyze Cldn7-and Cldn7*-containing model membranes. All buffers and solutions were freshly prepared and filtered (0.2 µm) before use. Freshly cleaved muscovite mica (12 mm diameter; Ted Pella Inc., grade V1) was mounted on a Teflon disc (16 mm) and washed with 3 × 50 µl of adsorption buffer (p-buffer + 50 mM Mg^2+^). Next, 50 µl proteoliposomes or liposomes (2 mM DOPC) were added to the mica surface and incubated for 15 min at RT. Unbound (proteo)liposomes were removed by washing the mica surface with 10 × 50 µl p-buffer. A drop of 20 µL buffer remained on the mica surface. The AFM measurements were performed using a Bruker (Billerica, MA, United States) Multimode 8 AFM with Nanoscope V Controller and J-scanner in the *ScanAsyst Fluid* mode*.* Bruker SNL-10 A cantilevers (Nominal parameters: 2 nm tip, resonance frequency f0 = 65 kHz, spring constant k = 0.35 N/m) were used. The scan rate was 1–10 Hz, the peak force amplitude 30–100 nm and the peak force tapping frequency 2–4 kHz. The scans were analyzed using the software NanoScope and Gwyddion (Nečas and Klapetek, 2012). The measured height-signal images were smoothed by removing a polynomial background, and scan rows were aligned using the *trimmed mean* method. The images were cropped to the area of interest and scaled to 2048 × 2048 px.

## 3 Results

### 3.1 Purified Human Cldn7 Oligomerizes in Detergent

**FIGURE 1:**
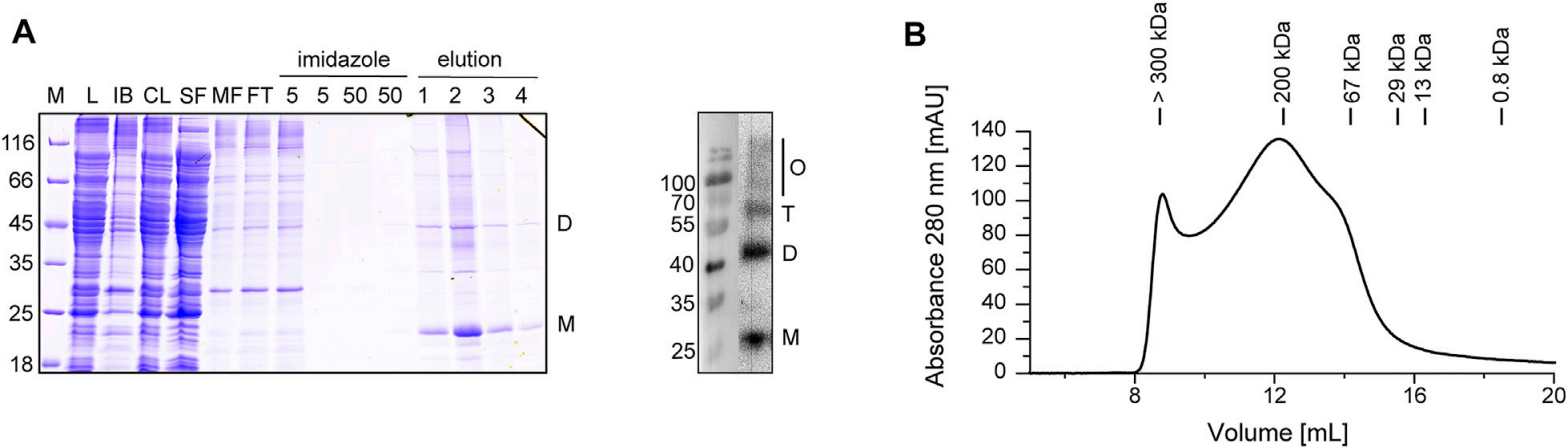
Purification and analysis of heterologously expressed Cldn7. **(A)** Purified Cldn7 was analyzed by SDS-PAGE (left) and Western blot analysis of the eluted protein (right). Samples of the following purification steps were analyzed: *E. coli* lysate (L), separated inclusion bodies/cell debris (IB), cleared lysate after centrifugation (CL), soluble protein fraction (SF), membrane protein fraction (MF), flow-through of IMAC (FT), washing steps (5 and 50 mM imidazole) and elution steps 1−4 (500 mM imidazole). The masses of the standards are given on the left in kDa. The position of the monomer (M), dimer (D), trimer (T) and higher oligomers (O) are labeled. **(B)** The oligomeric size of Cldn7 was analyzed by SEC, indicating formation of various Cldn7 oligomers.

Upon expression of a His-tagged Cldn7 in *E. coli* cells, *E. coli* membranes were solubilized with the detergent LDAO and Cldn7 was isolated via IMAC, resulting in a purified protein with ∼20 kDa (Figure 1A). The overall yield of purified Cldn7 was 1.8 mg/L cell culture. Using an anti-His-tag-antibody, bands with increased molecular masses were identified as SDS-stable Cldn7 oligomers. Monomers, dimers and trimers were clearly detectable, albeit also oligomers with higher molecular masses were detected, which, however, were not properly resolved anymore. Undoubtedly, there were still small impurities visible in the SDS-PAGE analysis. Since *E. coli* does not form tight junctions or septate junctions, these impurities very likely do not affect the functional analyses we performed in the following. Formation of higher-order oligomeric structures was further supported by SEC analyzes of Cldn7 in DDM-micelles (Figure 1B): while some protein was found in the void volume, representing aggregated or higher-order oligomeric species with molecular masses >300 kDa, two main peaks were resolved at 12.5 and 14 ml, which correspond to molecular masses of ∼200 and ∼65 kDa, respectively. Considering the calculated Cldn7 molecular mass of 26 kDa and the mass of a DDM micelle, which is 40–80 kDa (Green and Sambrook, 2012), the peaks likely correspond to a pentamer/hexamer and a monomeric Cldn7, respectively. Yet, the peaks are rather broad, and thus potentially represent multiple oligomeric species.

### 3.2 *In vitro* Analyses of Cldn7 *cis*-Interactions

Based on a recent analysis of the human Cldn15 structure, 6 residues were suggested to mediate *cis*-interactions of monomers within the membrane (Suzuki et al., 2014). The corresponding residues of human Cldn7 are depicted in Supplementary Figure S3A. To test whether these residues are also crucially involved in Cldn7 oligomerization, we mutated each residue individually to Ala as well as generated a Cldn7 variant in which all seven residues are mutated in parallel (Cldn7*).

We first aimed at monitoring the impact of (putatively) disturbed (*cis*-)oligomerization on the stability of human Cldn7 upon SDS-induced destabilization via measuring changes in the intrinsic Trp fluorescence. Despite the rather small changes in the average emission wavelength <λ>, a clear trend of decreasing <λ> with increasing χ_SDS_ was observed, until a plateau was reached at χ_SDS_ of 0.6 (Supplementary Figures S3B,C). Thus, a SDS-induced structural rearrangement of human Cldn7 can indeed be monitored via fluorescence spectroscopy. Yet, when the stability of the Cldn7* mutant was analyzed, the <λ> decrease was almost identical to the wt, and thus any potential impact of the mutations on the stability of Cldn7 cannot be observed via following Trp fluorescence.

**FIGURE 2:**
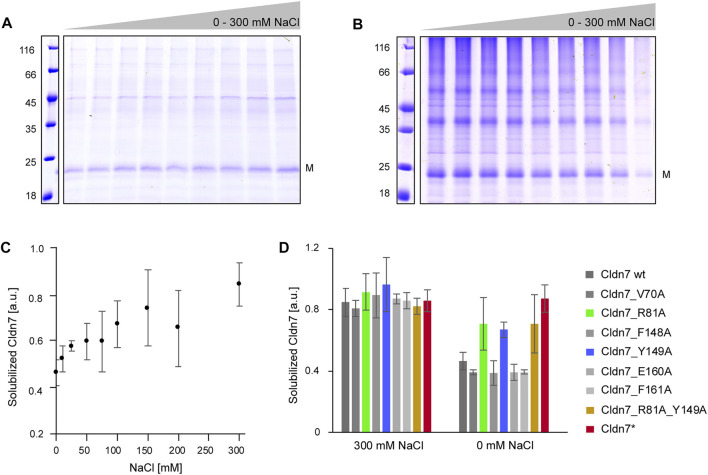
Increased ionic strength reduces Cldn7 *cis*-oligomerization. Cldn7 was solubilized in p-buffer with 0, 5, 25, 50, 75 100, 150, and 200 mM or 300 mM NaCl. After centrifugation, the supernatant (= solubilized Cldn7) and the pellet (= precipitated Cldn7) were analyzed by SDS-PAGE. The masses of the standards are given on the left in kDa. The position of the monomer (M) is labeled. **(A)** Representative SDS-PAGE analysis of solubilized Cldn7 samples in p-buffer with 0–300 mM NaCl. **(B)** Representative SDS-PAGE analysis of precipitated Cldn7 in p-buffer with 0–300 mM NaCl. **(C)** The fraction of solubilized Cldn7, calculated from the densiometrically determined intensities of the bands in **(A)** and **(B)**, increases with increasing NaCl-concentrations. (*n* = 3, error bars represent SD). **(D)** The solubilized fraction of Cldn7 wt and mutants were compared at 0 and 300 mM NaCl. At 0 mM NaCl, the fraction of solubilized Cldn7_R81A, Cldn7_Y149A, the double mutant Cldn7_R81A_Y149A and the Cldn7* mutant was clearly enhanced when compared to Cldn7 wt and the other Cldn7 mutants. (*n* = 9, error bars represent SD).

In our initial purification attempts we had observed that Cldn7 precipitates at NaCl concentrations below 200 mM, likely due to strong electrostatic interactions between the monomers. When we stepwise decreased the NaCl concentration from to 300 to 0 mM NaCl, Cldn7 interactions were disturbed and decreasing amounts of Cldn7 remained in solution after centrifugation, indicating that ions increase the interaction between Cldn7/detergents complexes (Figures 2A–C). Thus, this approach enabled us to quantify the amount of soluble Cldn7 at different NaCl concentrations and to compare the interaction propensity of Cldn7 wt with the interaction propensity of the mutants.

**FIGURE 3:**
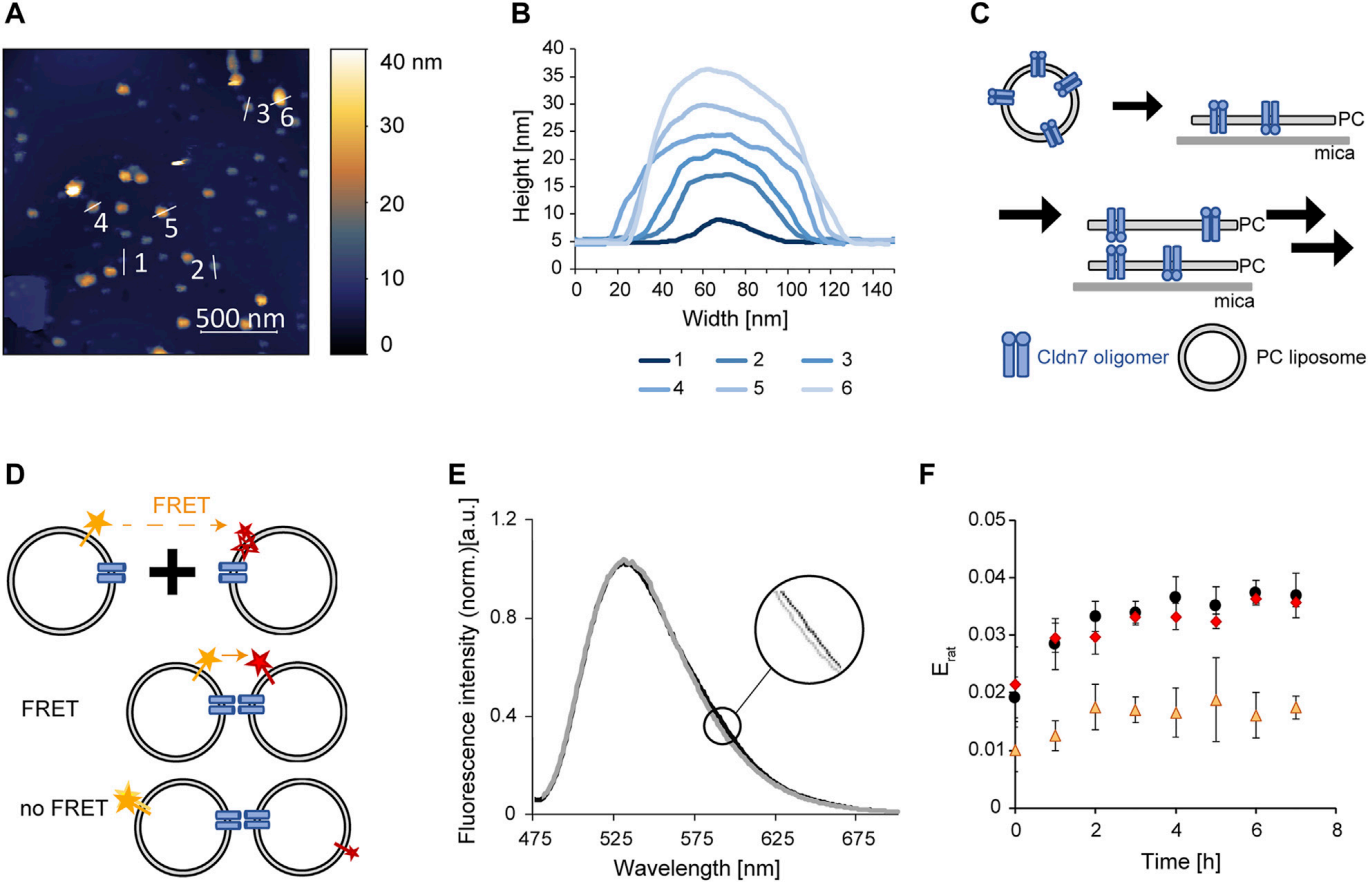
Trans-interaction of Cldn7 studied via AFM and FRET. **(A)** Typical AFM topography image showing Cldn7-proteoliposomes spread on a mica surface. Heights are indicated in the image using a false-color ruler. Profile lines indicate the position of the height analysis shown in **(B)**. **(B)** Height profile of spread Cldn7 proteoliposomes are shown. The data indicate formation of membrane stacks, as illustrated in **(C)**. **(C)** Cldn7 proteoliposomes likely spread on the mica surface and bind DOPC membrane patches via formation of Cldn *trans*-interactions. **(D)** FRET assay to measure interactions between Cldn-containing proteoliposomes. Note that only fluorophores close to Cldn interaction sites contribute to the FRET signal. Therefore, only minor changes were expected. **(E)** Fluorescence spectra were recorded upon excitation of the FRET donor dye NBD. In contrast to solely donor-labeled liposomes, a small increase of the LissRhod fluorescence at 590 nm was monitored. **(F)** FRET was analyzed by calculating the ratiometric FRET efficiency of control liposomes without Cldn7 (orange triangles), Cldn7 proteoliposomes (black circles) and Cldn7* proteoliposomes (red rhombus). In contrast to the liposome control, a larger E_rat_ and also a larger change of E_rat_ over 8 h was observed for proteoliposomes containing Cldn7 or Cldn7*. (*n* = 3, error bars represent SD).

As shown in Figures 3C,D, the amount of soluble protein increased by ∼ 50% at 300 mM NaCl compared to 0 mM. In contrast, no impact of the ionic strength was observed for Cldn7* where almost all protein was soluble at all NaCl concentrations. While most of the single mutants behaved like the wt, the impact of NaCl on the solubility of Cldn7_variants R81A and Y149A was lowered compared to the wt, indicating reduced inter-molecular interactions. Not surprisingly, also for an R81A_Y149A double mutant a similar behavior was observed. Thus, R81 and Y149 are likely involved in formation and/or stabilization of larger Cldn7 oligomers. As the corresponding residues mediate *cis*-contacts in case of Cldn15 (Suzuki et al., 2014), R81 and Y149 are likely responsible for Cldn7 *cis*-interactions.

### 3.3 Trans-interactions of Cldn7 Monomers

Next, we analyzed whether disturbed oligomerization of individual Cldn7 monomers affects *trans*-interaction of individual Cldn7 proteins, which are embedded in different membranes, as *e.g.* observed in tight junctions.

When we spread out Cldn7-containing proteoliposomes on a mica surface and analyzed the membrane via AFM, we observed spots with different diameters and heights. In fact, these spots are small, circular objects with diameters of 20–40 nm (Supplementary Figure S4). The rings had a height of about 5 nm at the outer rim, and a central hole. As Cdn7 monomers have a diameter of about 7 nm, these spots potentially represent Cldn7 oligomers, as observed for other Cldn`s and in line with the SEC analyses (Figure 1B), as further discussed below.

However, besides these circular Cldn7 oligomers, we also observed spots with much larger diameters, indicating the formation of larger assemblies (Figure 3A). Furthermore, spots with different heights were observed (Figure 3B). Yet, these spots always had a height of 5 nm or a multiple thereof. Since 5 nm is the height of the bilayer we determined for the protein-free solid-supported DOPC membrane, the spots likely represent stacked membranes with different amounts of stacked membrane patches (Figure 3B). Since no preferred orientation of Cldn7 monomers in the proteoliposomes was expected upon proteoliposome formation, Cldns of a single membrane can establish interactions in both directions (Figure 3C). The formation of membrane stacks was not affected by the NaCl concentration (Supplementary Figure S5), and also the Cldn7* mutant did form membrane stacks as the wt (Supplementary Figures S6, S7), indicating that the mutated residues are not required for establishing *trans*-interactions. Thus, this indicates that the intermolecular interactions described above indeed occur in *cis*. Nevertheless, as these measurements were qualitative, we next set up to establish a more quantitative assay to compare Cldn7-mediated membrane-tethering.

Therefore, we labeled a set of Cldn7-containing DOPC proteoliposomes with the lipid LissRhod-PE and a different fraction of proteoliposomes with the lipid NBD-PE. The dyes form a FRET pair and can transfer energy from the donor (NBD) to the acceptor (LissRhod) upon donor excitation. Thus, when *trans*-interaction of Cldn7 brings these two liposomes in close proximity, FRET is observable. Yet, the FRET signal was expected to be low as the FRET signal depends on the distance of the two fluorophores (Figure 3D). Even in linked liposomes, most labeled liposomes are not in sufficiently close distance to observe FRET. Consequently, liposome interaction mediated by Cldn7 solely slightly increases the probability of FRET between the differently labeled liposomes.

Indeed, the observed FRET signal was rather small (Figure 3E), but clearly increased with time after mixing of the liposomes (Figure 3F). Furthermore, the FRET signal observed with labeled proteoliposomes was at any time higher than the signal observed with labeled, Cldn7-free liposomes (Figure 3F). Nevertheless, the FRET intensities observed with the Cldn7 wt and the Cldn7* mutant were almost identical, again indicating that the disturbed oligomerization of Cldn7 (Figures 2C,D) does not dramatically affect its ability to interact in *trans*, in line with the results of the AFM analyses.

## 4 Discussion

Cldns oligomerize within the plasma membrane, and Cldn oligomers of adjacent cells interact to form tight-junctions. In fact, formation of human Cldn 1, 2, 3, 5, and 7 oligomers within a plasma membrane has previously been observed (Furuse et al., 1998; Coyne et al., 2003; Van Itallie and Anderson, 2006), which did, however, not allow detailed analyzes of the forces mediating protein oligomerization in a defined environment. However, recent attempt to analyze protein oligomerization *in vitro*, in a better defined environment, were not entirely conclusive: cell-free expressed, human Cldn2 and Cldn4 were shown to be multimeric in detergent-lipid mixed micelles, but not human Cldn1, 3 and 5 (Shinoda et al., 2016). Cldn4, which was heterologously expressed in Sf9 cells, appears to form hexamers when solubilized in perfluoro-octanoic acid (PFO), but not in DDM (Mitic et al., 2003; Belardi et al., 2019). These observations demonstrate already that the suitable *in vitro* preparation of Cldns for structural analysis is challenging. Furthermore, techniques (beyond SDS-PAGE and SEC analyses) allowing to experimentally study defined Cldn oligomerization *in vitro*, are rare.

In this study, we have successfully heterologously expressed and purified human Cldn7. The protein´s oligomeric structure was analyzed not only in detergent but also in a lipid bilayer environment, upon reconstitution into PC liposomes (Figure 3).

Purified human Cldn7 forms oligomeric structures in DDM (Figure 1B), which are even visible after SDS denaturation and PAGE analysis (Figure 1A). As only partial unfolding of Cldn7 was observed when the SDS-concentration was increased, the oligomers appear to be highly SDS-stable (Supplementary Figures S3B,C). In fact, SDS-stable oligomers were also observed recently, when human Cldn4 was heterologously expressed in Sf9 cells and solubilized by PFO (Mitic et al., 2003).

Formation of Cldn hexamers is discussed as an early step in the formation of tight junctions, based on the diameter of Cldn strands (Coyne et al., 2003; Findley and Koval, 2009). Thus, it is well possible that the oligomers identified here via SEC (Figure 1B) and AFM (Supplementary Figure S4) also represent stable hexameric Cldn7 assemblies. Based on our initial, low-resolution AFM analyses, these hexamers might be arranged ring-like within membranes with a central cavity (Supplementary Figure S4).

Based on structural and mutational analyses of human Cldn15 oligomerization, six residues were recently identified in the extracellular-localized head region that potentially establish inter-monomer *cis*-contacts (Suzuki et al., 2014). Noteworthy, also other residues may be involved in Cldn oligomerization. When the corresponding residues (Supplementary Figure S3A) were mutated in combination in Cldn7, we also observed disturbed formation of higher-order oligomers in presence of low NaCl concentrations (Figure 2). While Cldn7 in general precipitates at low NaCl concentration (Figures 2A–C), the Cldn7* mutant was not affected by a reduced ionic strength (Figure 2D). Yet, not all 6 residues contribute identically to Cldn7 oligomerization, and, based on our analyses, R81 and Y149 appear to be most crucial, at least in detergent (Figure 2D). In line with these observations, also in Cldn15, the residue corresponding to R81, R79, electrostatically interacts with E157, which corresponds to E160 of Cldn7 (Suzuki et al., 2014). Although we actually cannot finally discriminate between *cis-* and *trans*-interactions with the described experimental approaches (Figure 2), based on the analysis of Cldn15 it appears to be rather likely that these residues affect *cis*-oligomerization of Cldn7. Furthermore, these oligomers precipitate quickly at low speed, and thus, they must be rather large, which is difficult to obtain by *trans*-oligomerization of Cldn7 monomers or lower-order oligomers. Most likely, interactions of said residues result in formation of loose interactions between the detergent-embedded oligomers. In line with this assumption, Cldn7-mediated membrane-membrane interactions are not affected by reducing the NaCl concentration (Supplementary Figure S5). Additionally, the Cldn7* mutant still supports membrane-membrane interactions, as shown by AFM (Supplementary Figures S6, S7), indicating that the formation of stable, higher-order Cldn7 assemblies within the membrane might not be a prerequisite for linking two different membrane systems. Yet, ordered Cldn7 assembly likely is crucial for the formation of Cldn strands that polarize cells and form tight junctions.

In summary, we here established conditions for in-depth analyses of Cldn7 oligomerization within a membrane and the impact of this oligomerization on the formation of tight junctions. In the future, our experimental setup might allow analyzing not only isolated Cldn interactions, but also additional tight junction proteins, such as occludins, might be included in the analyses. The observations presented here indicate that Cldn7 *cis-* but not *trans-*interactions depend on electrostatic interactions involving the residues R81 and Y149.

## Data Availability

The original contributions presented in the study are included in the article/Supplementary Material, further inquiries can be directed to the corresponding author.
