# Supplementary material for: Human Claudin-7 cis-Interactions Are Not Crucial for Membrane-Membrane (Trans-) Interactions
Source: Front Mol Biosci. 2022 Jun 27;9:908383. doi: 10.3389/fmolb.2022.908383 (PMC9271825; doi:10.3389/fmolb.2022.908383)
Supplement: Supplementary file 1 [file DataSheet1.PDF]

Supporting Material

Figure S1: Primers used for site-directed mutagenesis

Mutation	Primer sequence	
**V70A**	fw	CAAAATGTACGACTCGGCGCTCGCCCTGTCCGCG
	rev	CGCGGACAGGGCGAGCGCCGAGTCGTACATTTTG
**R81A**	fw	GCCTTGCAGGCCACTGCGGCCCTAATGGTGGTC
	rev	GACCACCATTAGGGCCGCAGTGGCCTGCAAGGC
**F148A**	fw	CCATCAGATTGTCACAGACGCGTATAACCCTTTGATCCC
	rev	GGGATCAAAGGGTTATACGCGTCTGTGACAATCTGATGG
**Y149A**	fw	GCCATCAGATTGTCACAGACTTTGCGAACCCTTTGATCCCTACC
	rev	GGTAGGGATCAAAGGGTTCGCAAAGTCTGTGACAATCTGATGGC
**E160**	fw	CTACCAACATTAAGTATGCGTTTGGCCCTGCCATC
	rev	GATGGCAGGGCCAAACGCATACTTAATGTTGGTAG
**F161A**	fw	CAACATTAAGTATGAGGCGGGCCCTGCCATCTTTATTGG
	rev	CCAATAAAGATGGCAGGGCCCGCCTCATACTTAATGTTG
**F148A_Y149A**	fw	CCATCAGATTGTCACAGACGCGGCGAACCCTTTGATCCCTACC
	rev	GGTAGGGATCAAAGGGTTCGCCGCGTCTGTGACAATCTGATGG
**E160A_F161A**	fw	CTACCAACATTAAGTATGCGGCGGGCCCTGCCATCTTTATTGG
	rev	CCAATAAAGATGGCAGGGCCCGCCGCATACTTAATGTTGGTAG

Figure S2: Extinction coefficients used for protein concentration determination

Protein	MW [kDa]	Extinction coefficient ε [L mol^{l}1 cm^{-1}]
**Cldn7**	26.3	48400
**Cldn7_V70A**	26.3	48400
**Cldn7_R81A**	26.3	48400
**Cldn7_F148A**	26.3	48400
**Cldn7_Y149A**	26.3	46910
**Cldn7_E160A**	26.3	48400
**Cldn7_F161A**	26.3	48400
**Cldn7_R81A_Y149A**	26.2	46910
**Cldn7***	25.9	46910

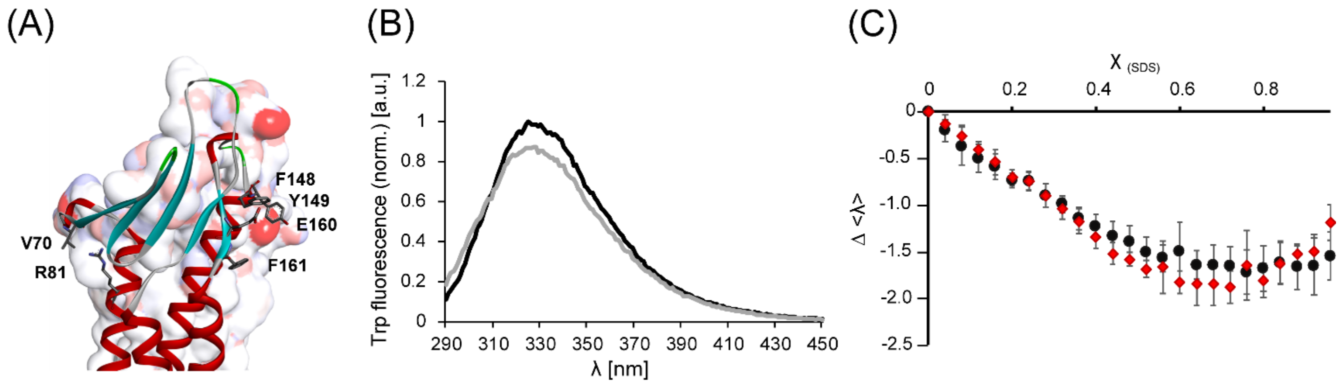

Figure S3: SDS-induced alterations of the Cldn7 structure monitored via Trp fluorescence changes

A: V70, R81, F148, Y149, E160 and F161 are potentially relevant for Cldn7 oligomerization within a membrane (Suzuki et al., 2014). The position of these amino acids in the region of ECL 1 and 2 is highlighted in a Cldn7 structure modeled using AlphaFold (Jumper et al., 2021; Varadi et al., 2022). B: SDS-induced Cldn7 unfolding was followed via monitoring Trp fluorescence in micelles containing χSDS = 0 (black line) to χSDS = 0.95 (grey line). <λ> was shifted to shorter wavelengths with increasing SDS fractions. C: The shift of <λ> upon SDS-induced unfolding of Cldn7* (red rhombus) differs only slightly from the <λ> determined using Cldn7 wt (black circles). (n=9, error bars represent SD).

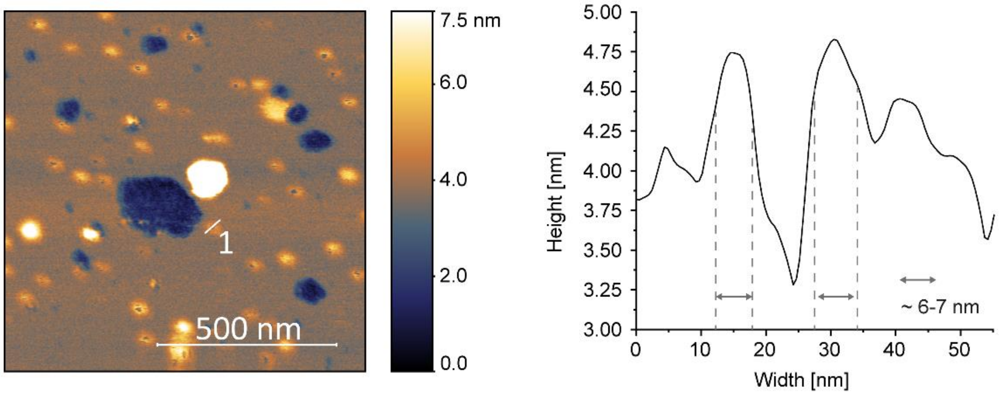

Figure S4: AFM of Cldn7 proteoliposomes

Example of an AFM topography image showing spread Cldn7 proteoliposomes in p-buffer. Heights are indicated in the image using a false-color ruler. The profile line indicates the position of the height analysis shown on the right.

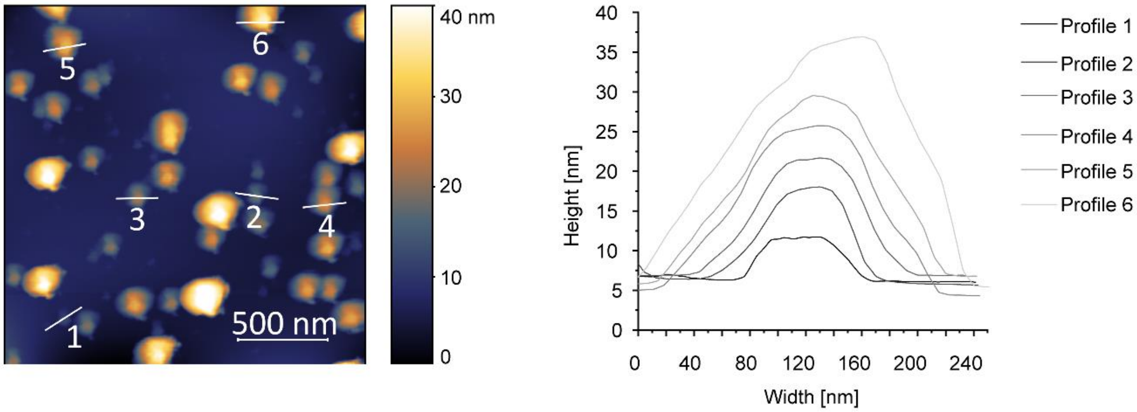

Figure S5: AFM of Cldn7 proteoliposomes in absence of NaCl

AFM topography image showing spread Cldn7 proteoliposomes in p-buffer without NaCl. Heights are indicated in the image using a false-color ruler. Profile lines indicate the positions of the height analyses shown on the right.

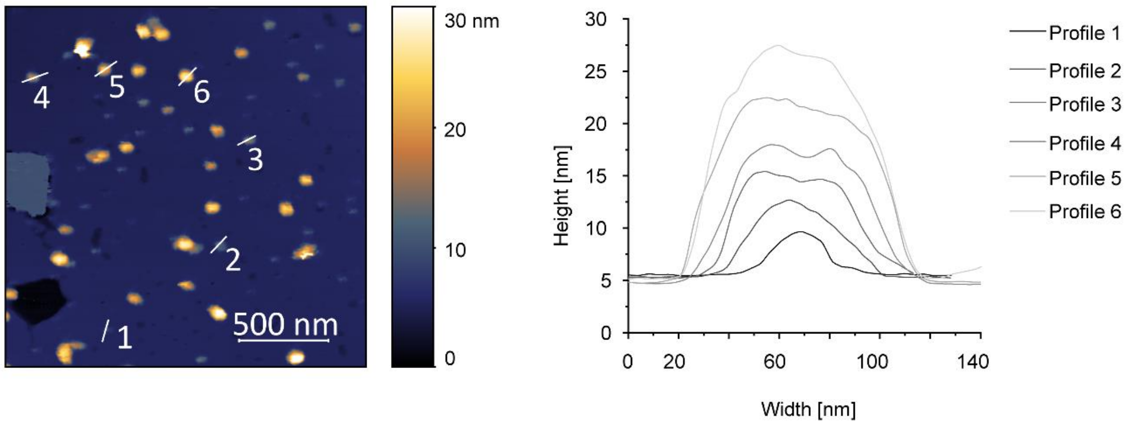

Figure S6: AFM of Cldn7* proteoliposomes

AFM topography image showing spread Cldn7* proteoliposomes in p-buffer. Heights are indicated in the image using a false-color ruler. Profile lines indicate the positions of the height analyses shown on the right.

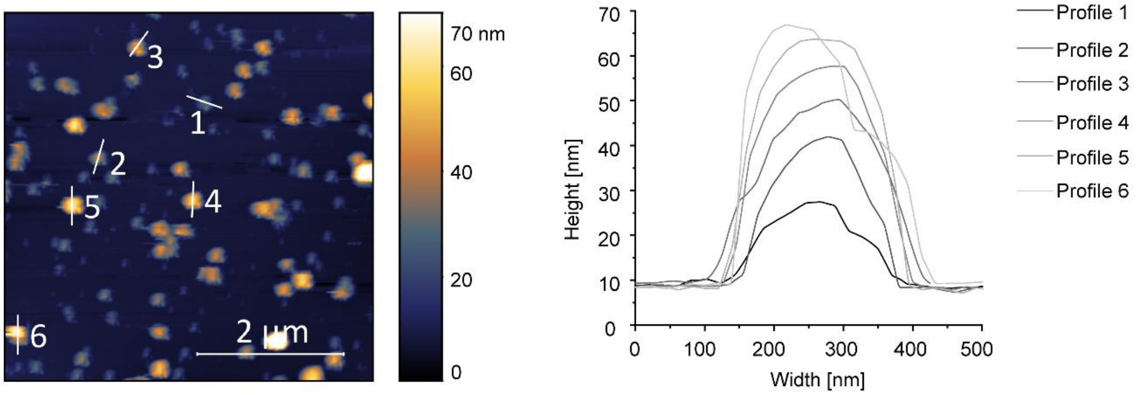

Figure S7: AFM of Cldn7* proteoliposomes in absence of NaCl

AFM topography image showing spread Cldn7 proteoliposomes in p-buffer without NaCl. Heights are indicated in the image using a false-color ruler. Profile lines indicate the positions of the height analyses shown on the right.
